# Inaccessible Biodiversity on Limestone Cliffs: *Aster tianmenshanensis* (Asteraceae), a New Critically Endangered Species from China

**DOI:** 10.1371/journal.pone.0134895

**Published:** 2015-08-26

**Authors:** Guo-Jin Zhang, Hai-Hua Hu, Cai-Fei Zhang, Xiao-Juan Tian, Hui Peng, Tian-Gang Gao

**Affiliations:** 1 State Key Laboratory of Systematic and Evolutionary Botany, Institute of Botany, the Chinese Academy of Sciences, Beijing, 100093, China; 2 University of the Chinese Academy of Sciences, Beijing, 100039, China; 3 Tianmenshan National Forest Park, Zhangjiajie, Hunan, 427300, China; National Cheng-Kung University, TAIWAN

## Abstract

*Aster tianmenshanensis* G. J. Zhang & T. G. Gao, a new species of Asteraceae from southern China is described and illustrated based on evidence from morphology, micromorphology and molecular phylogeny. The new species is superficially similar to *Aster salwinensis* Onno in having rosettes of spatulate leaves and a solitary, terminal capitulum, but it differs by its glabrous leaf margins, unequal disc floret lobes and 1-seriate pappus. The molecular phylogenetic analysis, based on nuclear sequences ITS, ETS and chloroplast sequence *trnL-F*, showed that the new species was nested within the genus *Aster* and formed a well supported clade with *Aster verticillatus* (Reinw.) Brouillet *et al*. The new species differs from the latter in having unbranched stems, much larger capitula, unequal disc floret lobes, beakless achenes and persistent pappus. In particular, *A*. *tianmenshanensis* has very short stigmatic lines, only ca. 0.18 mm long and less than 1/3 of the length of sterile style tip appendages, remarkably different from its congeners. This type of stigmatic line, as far as we know, has not been found in any other species of *Aster*. The very short stigmatic lines plus the unequal disc floret lobes imply that the new species may have a very specialized pollination system, which may be a consequence of habitat specialization. The new species grows only on the limestone cliffs of Mt. Tianmen, Hunan Province, at the elevation of 1400 m. It could only be accessed when a plank walkway was built across the cliffs for tourists. As it is known only from an area estimated at less than 10 km^2^ and a walkway passes through this location, its habitat could be easily disturbed. This species should best be treated as Critically Endangered based on the International Union for Conservation of Nature Red List Categories and Criteria B2a.

## Introduction

*Aster*, the type genus of Asteraceae, contains approximately 152 species widely distributed in Eurasia [1]. The circumscription of the genus has been changed greatly since the 1990s. In a broad and traditional concept, the genus contains about 180–1000 species widely distributed in the Northern Hemisphere [2–5]. The North American *Aster* species, except for *Aster alpinus* L., have been split into more than 10 independent genera [4, 6–7] based mainly on morphology and cytology. This is also supported by phylogenetic analysis based on ITS data [8]. The *Aster* species from Africa, which were tentatively treated as an uncertain group by Nesom [4], were shown to be a quite different group based on molecular evidence [9]. They were transferred to a new genus *Afroaster *[10]. The remaining Eurasian *Aster* complex includes many small segregate genera, such as *Doellingeria*, *Kalimeris*, *Heteropappus*, *Miyamayomena*, and *Rhynchospermum*, which were established based on one or a very few morphological characters. Recent molecular phylogenetic analysis [9, 11–12] showed that many of these segregate genera were nested within *Aster* and should be included in the genus. However, the relationships between some other genera and *Aster* are still in dispute and some of them probably will have to be included within *Aster* [1]. Further molecular phylogenetic studies with more sampling, more markers and detailed morphological studies of the *Aster* complex are needed.

During the course of revising the genus *Aster* of the world, a morphologically remarkable species of *Aster* from southern China was encountered. It was located in Mt. Tianmen National Forestry Park, Zhangjiajie City, Hunan Province, China, where it grew on the steep limestone cliffs that are difficult to get access to until a plank walkway was constructed in 2006. The species is superficially very similar to *Aster salwinensis*, but after careful observations of evidence from morphology, micromorphology and molecular phylogeny, we conclude it as a new species that is distinct from *A*. *salwinensis*.

## Materials and Methods

### Ethics statement

The collecting location reported in this work is in charge by the Mt. Tianmen National Forestry Park, which permitted our research here. We collected the plants in the company of their staff (XJT and HP). The species described here is currently not included in the Chinese Red Data Book.

### Taxon sampling, DNA extraction, PCR reaction and sequencing

**Fig 1:**
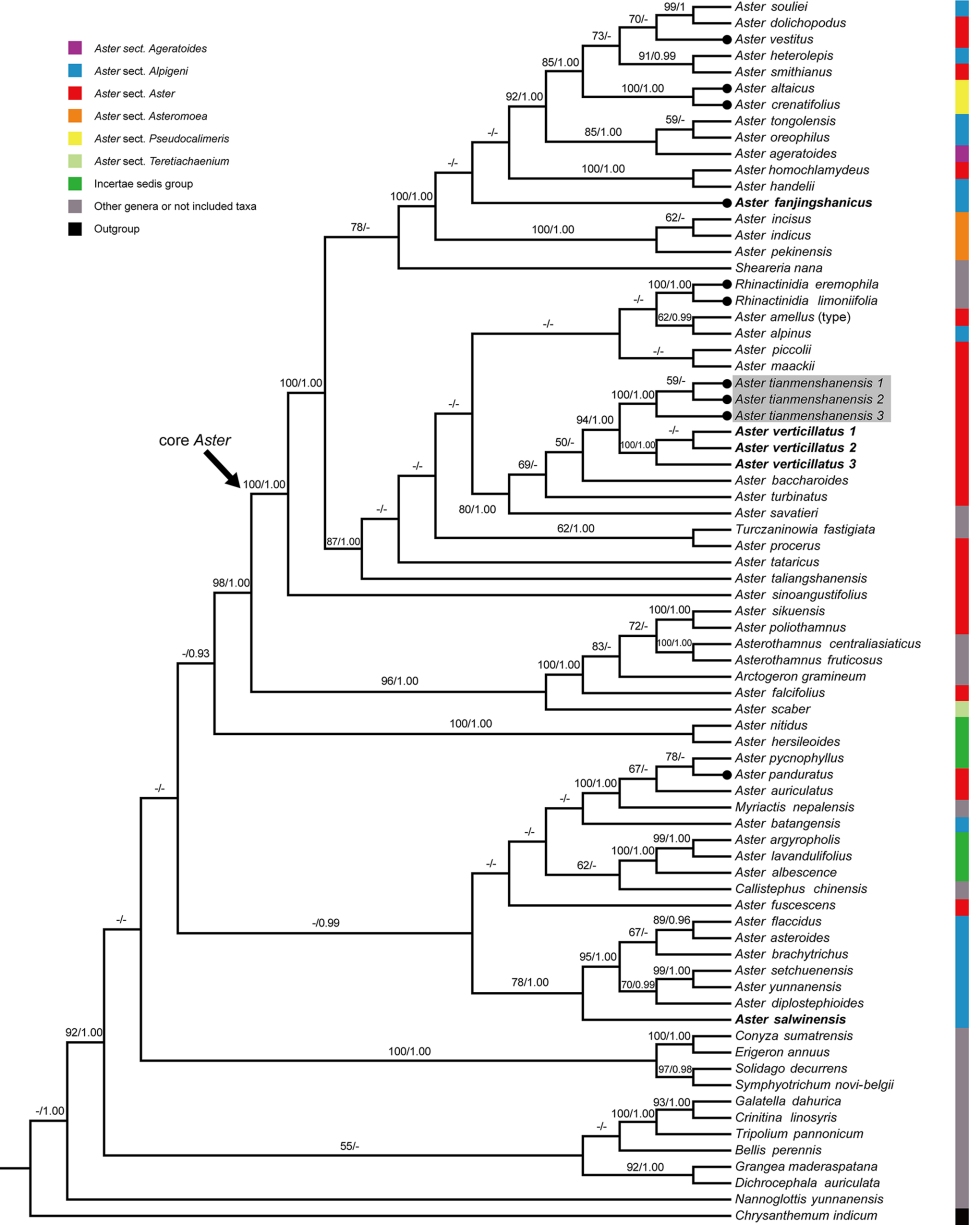
The cladogram of the maximum likelihood (ML) tree from the combined data (ITS, ETS and *trnL-F*), showing the position of *Aster tianmenshanensis*. Values above branch represent bootstrap values (BS) for maximum likelihood and Bayesian posterior probabilities (PP) respectively; the dash (–) indicates BS < 50% or PP < 0.90. The new species is indicated by shading and its putative relatives in bold. Taxonomic treatments in *Flora of China* are illustrated with color bars. The type species *Aster amellus* is indicated in brackets. Species with unequal lobes are shown with black dots.

We downloaded the ITS, ETS and *trnL-F* sequences of 62 species from GenBank, representing 19 genera and major clades of the genus *Aster* and its relatives [9, 12]. In this study we newly sequenced nine additional species including three samples of the new taxon *Aster tianmenshanensis* and three samples of its putative relative *Aster verticillatus* (Reinw.) Brouillet, Semple & Y. L. Chen. We followed the treatments and names in *Flora of China* [1]. For ease of discussion, we collectively call all the *Aster* species at and above the species *Aster sinoangustifolius* Brouillet, Semple & Y. L. Chen in Fig 1 “core *Aster*” which forms a strongly supported clade and includes the type species of the genus, i.e. *Aster amellus* L. The *Aster* species in this clade plus other *Aster* species below this clade are called *Aster*. The other *Aster* species may represent independent genera different from the core *Aster*, which requires more study to clarify. In the phylogenetic analysis, we selected *Chrysanthemum indicum* L. as outgroup following previous work [12]. Voucher specimens for newly sequenced materials were deposited in PE. Voucher information and GenBank accession numbers are listed in S1 Table.

Leaf tissue was collected in the field and dried in silica gel. Total template DNA was extracted using the CTAB Plant Genomic DNA Kit (DN14) (Biomed Co., Beijing). PCR amplification of the ITS and ETS followed Linder et al. [13] or minor modifications thereof. The ETS region was amplified using Ast-8 [14] and 18S-IGS [15] while the ITS region was amplified using ITS4 [16] and ITS5 [17] for forward and reverse primers. The *trnL-F* region was amplified with the “c” and “f” primers of Taberlet et al. [18] with the parameters 94°C, 3 min; 35 × (94°C, 1 min; 52°C, 1 min; 72°C, 2 min); 72°C, 7 min. PCR was performed using Veriti 96 well thermal cycler (Applied Biosystems, USA) in 25 μL volumes. Each reaction contained with 1 μL template DNA (~ 50 ng DNA), 12.5 μL 2 × Taq PCR Mastermix (Biomed, China), 2.5 μL of each primer (1 μM) and 7 μL ddH_2_O. Purification of the PCR products and sequencing were performed by Biomed Co., Beijing.

### Data analysis

All the DNA sequences were aligned by Clustal X 1.83 [19], and manually adjusted using BioEdit 7.0.8.0 [20].

The optimal model of DNA substations was selected using the Akaike information criterion [21] as applied in jModelTest 2.1.4 [22] prior to the maximum likelihood (ML) analyses and Bayesian inference (BI). The GTR+G model was indicated as best fit for ETS and ITS, and TVM+G for *trnL-F*. Phylogenetic analyses were then conducted for individual data sets and concatenated data matrix. ML analyses were inferred in RAxML 8.0.24 [23] using 1000 bootstrap replicates under the GTRGAMMA to get bootstrap values (BS) of each node. The BI analyses were performed using MrBayes 3.2.2 [24] employing the estimated models. Four chains, each starting with a random tree, were run for 2,000,000 generations with trees sampled every 1000 generations. Bayesian posterior probabilities (PP) were calculated from the majority consensus of all sampled trees after discarding the first 500 (25%) trees as the “burn-in”. ML and BI analyses were all implemented on the CIPRES science gateway portal [25]. Before the datasets combination, the incongruence length difference test [26] was performed on PAUP* v.4.0b10 [27].

### Morphological observations

The description and the line drawing of the new species *Aster tianmenshanensis* were based on examination under a stereomicroscope of living material and dry specimens. The measurements were based on living and FAA-fixed materials. The morphological comparison with other species of *Aster* was based on study of herbarium specimens from PE (Chinese National Herbarium, Institute of Botany, the Chinese Academy of Sciences).

### Micromorphological observations

Floral micromorphological observations were conducted on the new species and its putative relatives (*Aster salwinensis* Onno, *Aster fanjingshanicus* Y. L. Chen & D. J. Liu and *Aster verticillatus* (Reinw.) Brouillet, Semple & Y. L. Chen). The source of this material is listed in S1 Table. For sectioning techniques, we basically followed Lewis’s method [28]. Capitula from herbarium specimens were soaked in FAA solution for 24 hours. All sampled materials were then cleaned using a supersonic generator for 5 minutes, 100 Hz and treated in 5% NaOH (corolla and style for 6 hours, anther for 12 hours). After rinsing with water, the material was mounted on slides, and flooded with Hoyer’s solution. Samples were then examined using light microscopy and photographed. The disc floret corolla, filament collar, anther base, anther tip appendage, thickening pattern of anther endothecial tissue, stylopodium, stigmatic lines (where pollens germinate) and sterile style tip appendages were all observed under a light microscopy and photographed using Leica DM5000B.

## Results

### Molecular phylogenetic analysis

Results from the incongruence length difference (ILD) test between the ITS + ETS and *trnL-F* data sets showed no obvious conflict existed (P = 0.01). Strongly supported incongruence for conflicting nodes was not found between trees obtained from individual data sets (here considered BS ≥ 85% and PP ≥ 0.95). Thus, data sets were combined. The ITS, ETS and *trnL-F* contained 661, 540 and 945 characters respectively, and the combined dataset consisted of 2146 aligned characters with 324 parsimony-uninformative variable characters and 524 parsimonious informative characters. Consensus trees from BI analyses had nearly identical topologies to the ML tree. The best ML tree (-InL = -15917.400943) is presented in Fig 1.

The molecular evidence showed that three samples of *Aster tianmenshanensis* were grouped together with strong support (BS = 100%, PP = 1.00) and was nested within the core *Aster* clade (BS = 100%, PP = 1.00). Within the core *Aster* clade, the new species formed a well supported subclade with three samples of *Aster verticillatus* (in *Aster* sect. *Aster* in *Flora of China*) (BS = 94%, PP = 1.00) (Fig 1). *Aster fanjingshanicus* (in *Aster* sect. *Alpigeni* Nees in *Flora of China*), a morphologically similar species to the new species was located in a different subclade within the core *Aster* clade. This well supported subclade (BS = 100%, PP = 1.00) consisted of *Aster fanjingshanicus* and *A*. *pekinensis* (Hance) F. H. Chen, *A*. *handelii* Onno, *A*. *ageratoides* Turcz., *A*. *crenatifolius* Hand.-Mazz., *A*. *smithianus* Hand.-Mazz., *A*. *vestitus* Franch. *et al*. (Fig 1). *Aster salwinensis* (in *Aster* sect. *Alpigeni* in *Flora of China*), the superficially most morphologically similar species to the new species, however, was located outside of the core *Aster* clade. It formed a moderately supported clade with *Aster asteroides* (DC.) Kuntze, *A*. *flaccidus* Bunge, *A*. *brachytrichus* Franch., *A*. *yunnanensis* Franch., *A*. *setchuenensis* Franch. and *A*. *diplostephioides* (DC.) Benth. ex C. B. Clarke (BS = 78%, PP = 1.00) (Fig 1).

### Morphological observations

**Fig 2:**
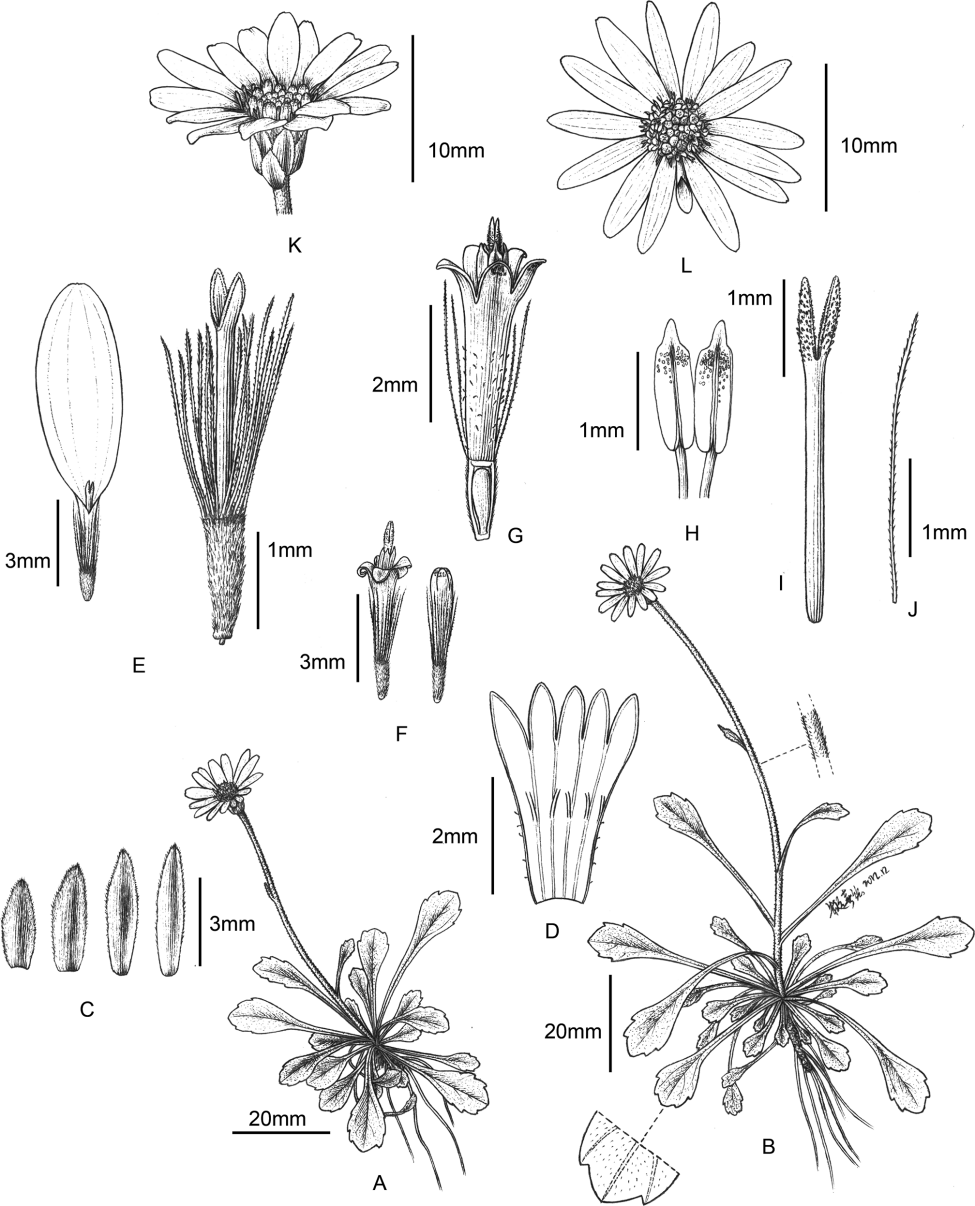
*Aster tianmenshanensis*. (A–B) habit. (C) phyllaries. (D) disc floret corolla, inside view, stamens removed. (E) ray florets, corolla removed in the right one. (F–G) disc florets. (H) stamens. (I) style of disc floret. (J) barbellate bristle. (K–L) capitula, lateral (K) and top (L) views. Drawn by Y. X. Zhu from *C*. *F*. *Zhang* 2718.

**Fig 3:**
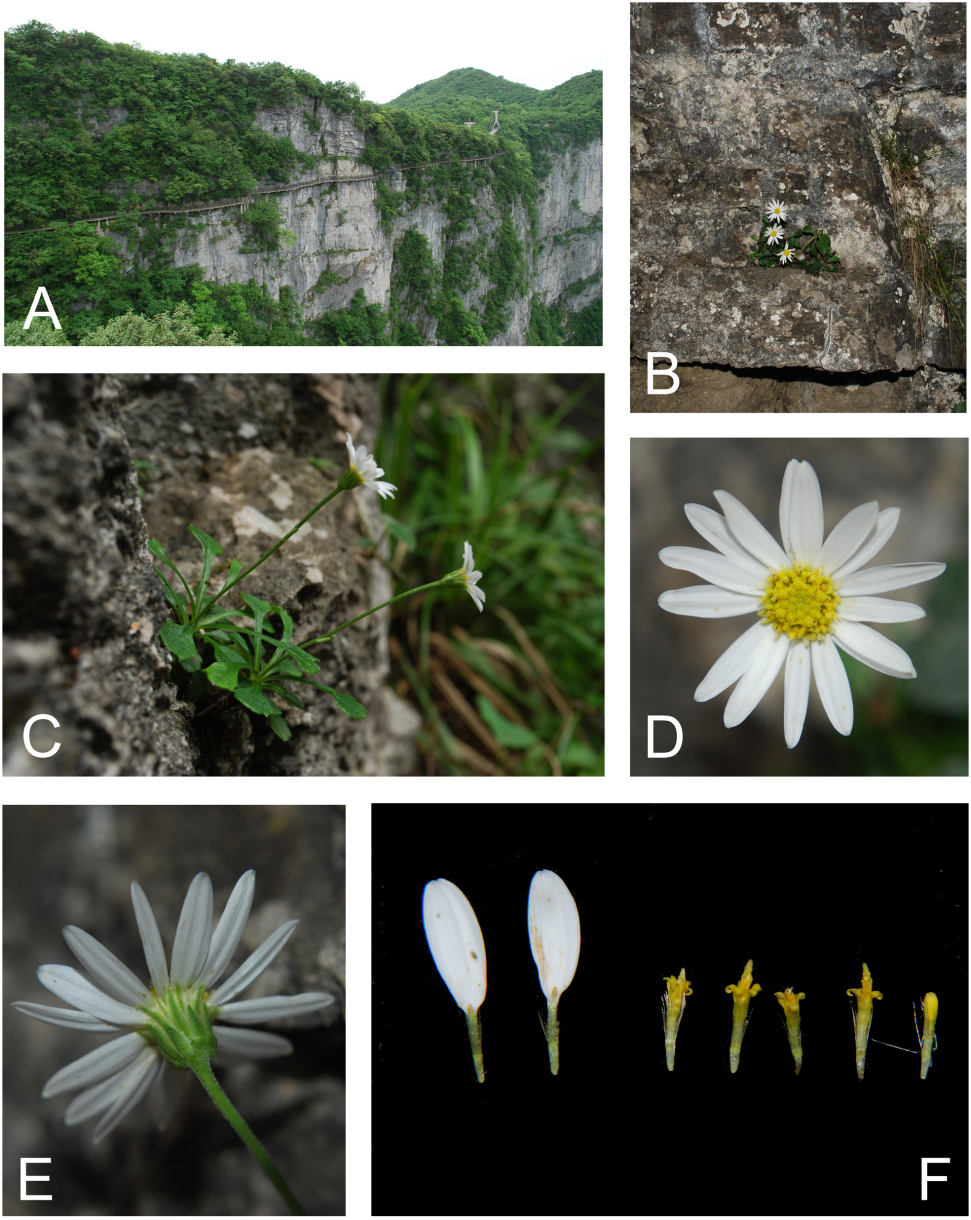
Habitat and morphology of *Aster tianmenshanensis*. (A–B) habitat. (C) individuals in flowering. (D) capitulum, top view. (E) capitulum, lateral view, showing the involucre. (F) ray and disc florets.

*Aster tianmenshanensis* is superficially most similar to *Aster salwinensis* (in *Aster* sect. *Alpigeni* in *Flora of China*) by its rosette leaves and solitary, terminal capitulum, while differs from the latter by its glabrous leaf margins (versus ciliate in *A*. *salwinensis*), unequal disc floret lobes (versus equal), and 1-seriate pappus (versus 4-seriate) (Fig 2A, 2B, 2D, 2E and 2G; Fig 3B, 3C, 3D, 3E and 3F).

*Aster tianmenshanensis* is also similar to *Aster fanjingshanicus* (in *Aster* sect. *Alpigeni* in *Flora of China*) by its rosette leaves and solitary, terminal capitulum, while differs from the latter by its glabrous leaf margins (versus ciliate in *A*. *fanjingshanicus*), glabrous to shortly pubescent stems and leaves (versus villous), unequal phyllaries (versus equal) and white pappus (versus brownish) (Fig 2A, 2B, 2D, 2E, 2G and 2K; Fig 3C, 3E and 3F).

The new species is also similar to its phylogenetically most closely related species *Aster verticillatus* (in *Aster* sect. *Aster* in *Flora of China*) by the imbricate, 2–3 seriate phyllaries, scarious phyllaries margins, and glandular disc floret corollas, but differs from that species in having unbranched aerial stems, larger capitula (15–20 mm in diameter) rather than smaller capitula (4–5 mm in *A*. *verticillatus*), beakless achenes rather than beaked ones, and in having persistent pappus rather than caducous and often absent pappus (Fig 2A, 2B, 2D, 2E, 2G, 2K and 2L; Fig 3C, 3D and 3F).

In the taxonomy of *Aster* and its close relatives, unequal lobes of the disc floret corolla used to be a diagnostic character for the genera *Heteropappus* Less. (= *Aster* sect. *Pseudocalimeris* Kitam. in *Flora of China*) and *Rhinactinidia* Novopokr. However, some species of *Aster*, such as *A*. *vestitus*, *A*. *panduratus* Nees ex Walp., which were regarded as having equal lobes in the literature [1, 3], have proved to have unequal lobes when more carefully examined (Fu, pers. comm.). In addition, as shown in the phylogenetic tree (Fig 1), species with unequal lobes are found in distantly related clades. Such similarity might be the result of convergent evolution.

### Micromorphological observations

**Fig 4:**
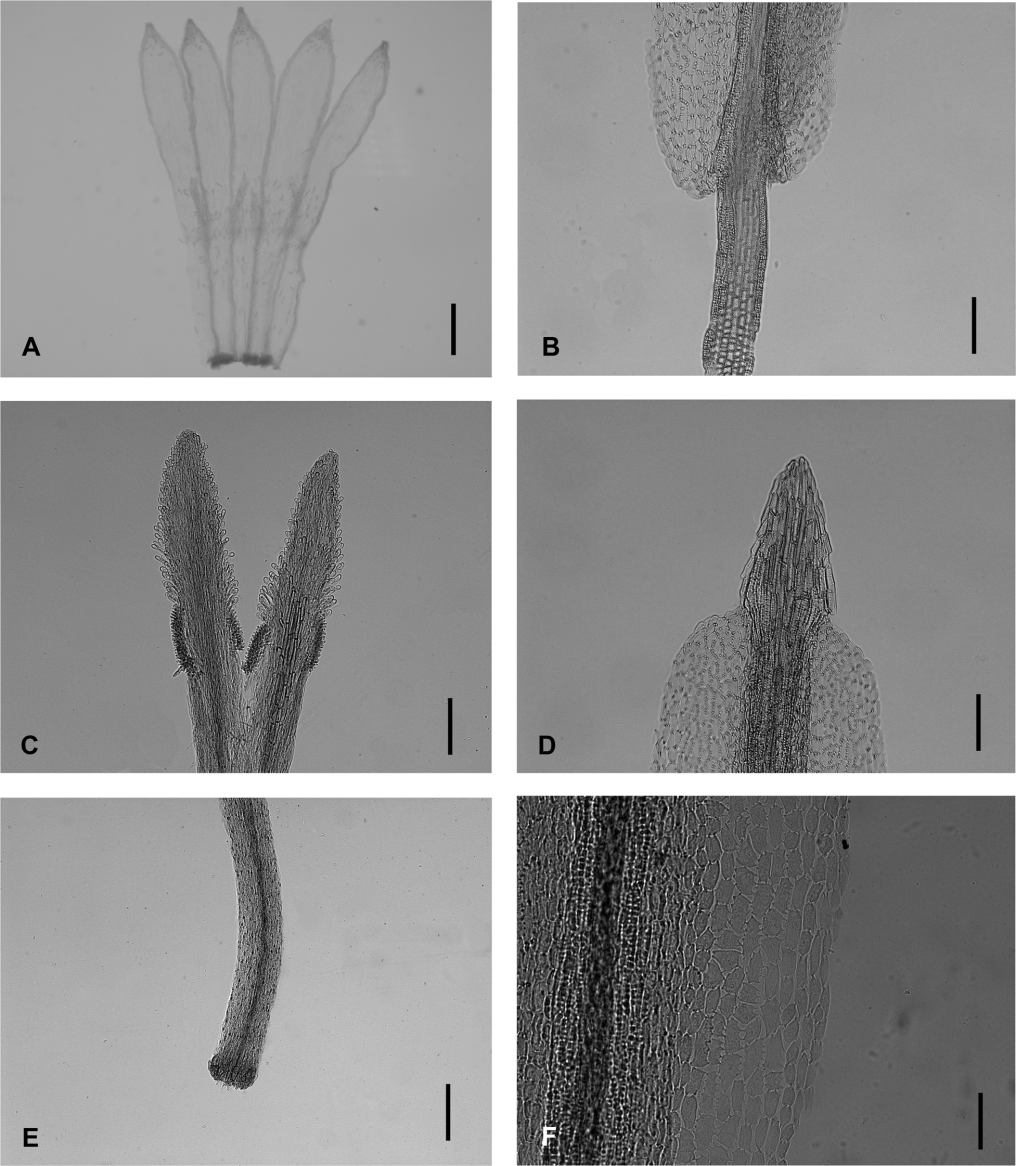
Micromorphology of *Aster tianmenshanensis*. (A) disc floret corolla. (B) anther base appendage and filament collar. (C) style branches and stigmatic lines. (D) anther tip appendage. (E) stylopodium. (F) anther endothecial tissue. Scale bars, 500 μm (A); 100 μm (B, D); 200 μm (C, E); 50 μm (F).

**Fig 5:**
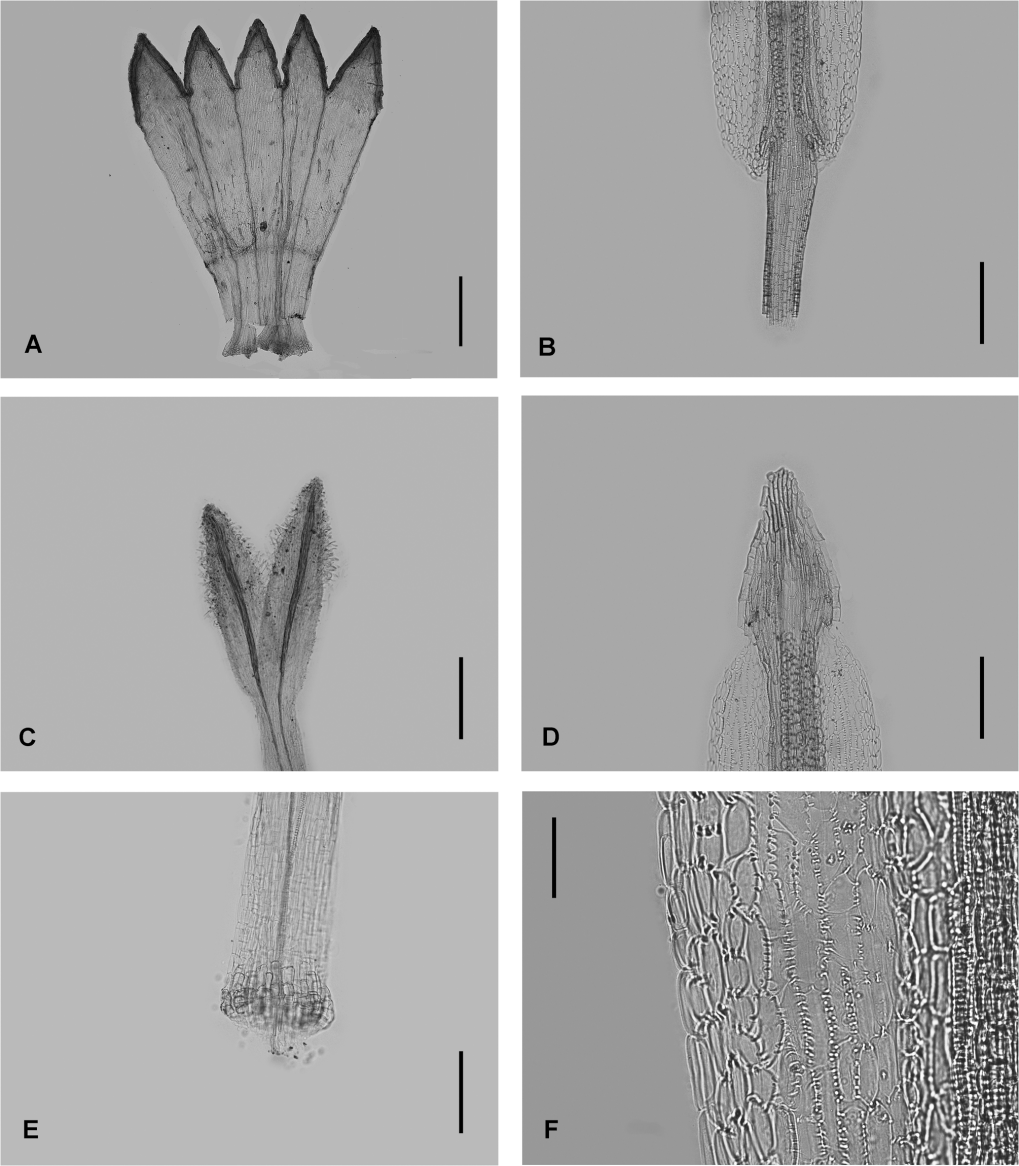
Micromorphology of *Aster salwinensis*. (A) disc floret corolla. (B) anther base appendage and filament collar. (C) style branches and stigmatic lines. (D) anther tip appendage. (E) stylopodium. (F) anther endothecial tissue. Scale bars, 1 mm (A); 200 μm (B, D, E); 500 μm (C); 100 μm (F).

The floral micromorphology of *Aster tianmenshanensis* is similar to *Aster salwinensis* in having an unexpanded filament collar (cylindrical type), obtuse anther base, triangulate anther tip appendage, anther endothecial tissue with polarized thickened middle cells and radially thickened lateral cells (radial and polarized types) and unexpanded stylopodium (Figs 4 and 5). The new species, however, is different from *A*. *salwinensis* by its unequal corolla lobes (Fig 4A) and very short stigmatic lines (only ca. 0.18 mm long, less than 1/3 of the length of sterile style tip appendages) (Fig 4C). *A*. *salwinensis* has equal disc floret corolla lobes, and much longer stigmatic lines (ca. 0.6 mm long and only slightly shorter than the sterile style tip appendage) (Fig 5A and 5C).

**Fig 6:**
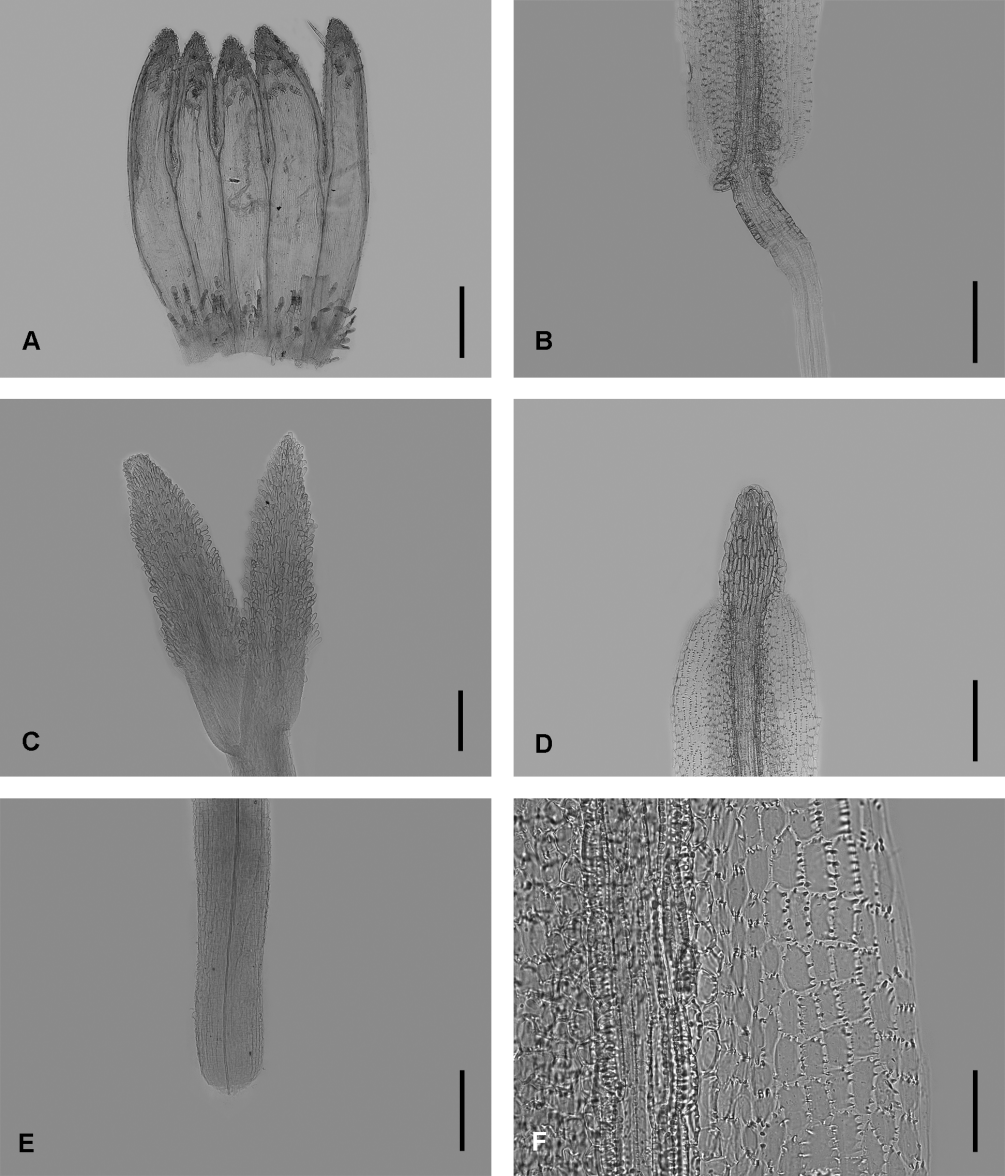
Micromorphology of *Aster fanjingshanicus*. (A) disc floret corolla. (B) anther base appendage and filament collar. (C) style branches and stigmatic lines. (D) anther tip appendage. (E). stylopodium. (F) anther endothecial tissue. Scale bars, 500 μm (A); 200 μm (B, C, D, E); 50 μm (F).

*Aster tianmenshanensis* is also similar to *Aster fanjingshanicus* in unexpanded filament collar, obtuse anther base, triangulate anther tip appendage, anther endothecial tissue with polarized thickened middle cells and radially thickened lateral cells (radial and polarized types) and unexpanded stylopodium (Figs 4 and 6), while differs from the later by its shorter stigmatic line (0.18 mm versus 0.31 mm in *A*. *fanjingshanicus*) (Figs 4C and 6C) and only glandular (versus glandular and pilose) apex of disc floret corolla lobes (Figs 4A and 6A).

**Fig 7:**
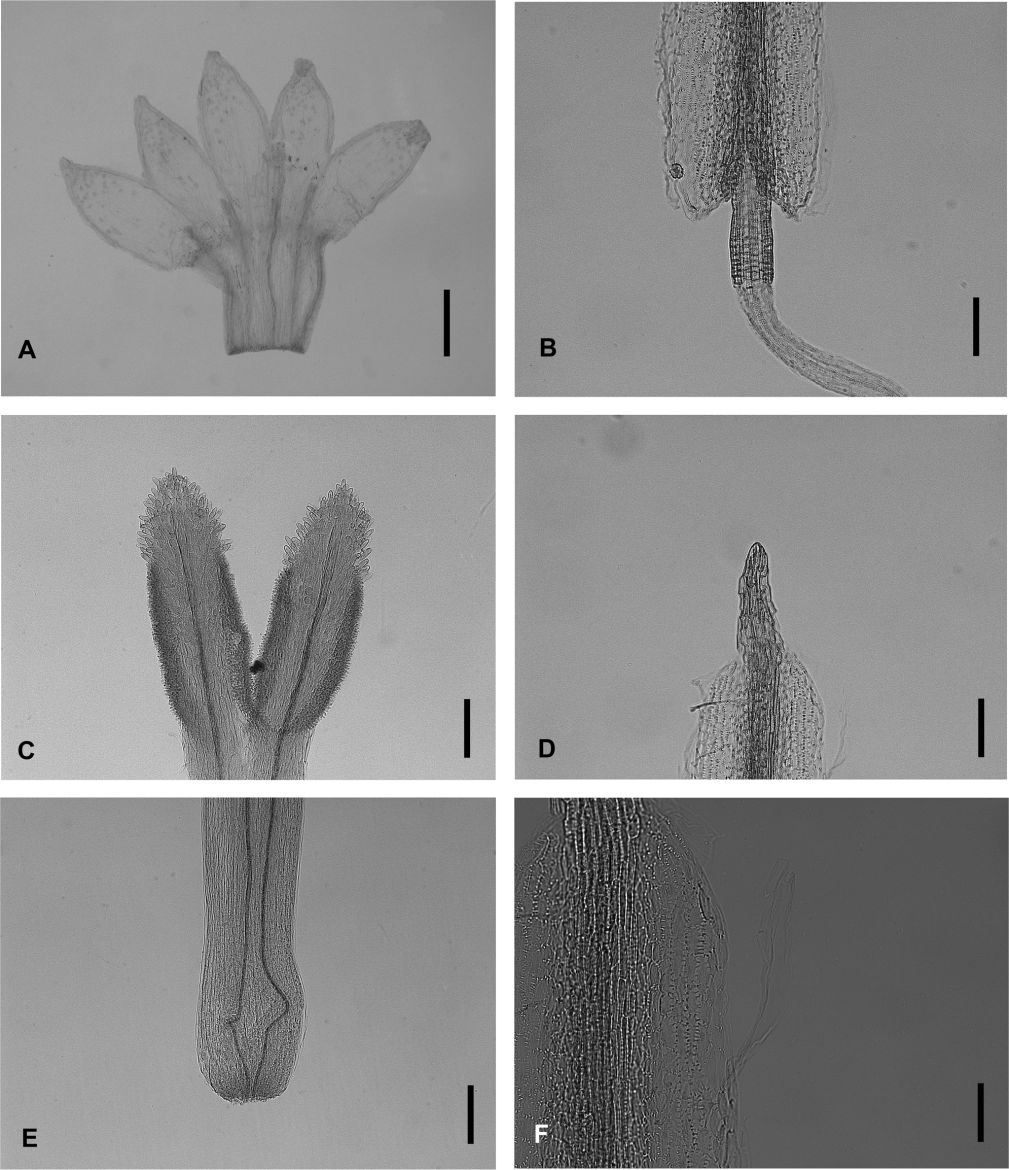
Micromorphology of *Aster verticillatus*. (A) disc floret corolla. (B) anther base appendage and filament collar. (C) style branches and stigmatic lines. (D) anther tip appendage. (E) stylopodium. (F) anther endothecial tissue. Scale bars, 500 μm (A); 100 μm (B, D); 200 μm (C, E); 50 μm (F).

*Aster tianmenshanensis* is different from *Aster verticillatus* in corolla lobes and stigmatic lines discussed above (Figs 4 and 7). *Aster verticillatus* has equal (versus unequal) disc floret corolla lobes and much longer stigmatic lines (ca. 0.6 mm long and nearly as 3 times longer as the sterile style tip appendage) (Figs 4A, 4C and 7A, 7C).

Very short stigmatic lines plus unequal disc florets lobes, these two characters of *A*. *tianmenshanensis* are rare and remarkable within the genus *Aster*. These indicate that the new species may have a specialized pollination system.

## Conclusion and Discussion

### Taxonomic treatment

***Aster tianmenshanensis* G. J. Zhang & T. G. Gao, sp. nov.** [urn:lsid:ipni.org: names: 77148277–1]

**Type.** CHINA. Hunan Province, Zhangjiajie City, Mt. Tianmen, alt. 1400 m, 110° 28´ E, 29° 3´ N, 2 June 2010, *C*. *F*. *Zhang 2718* (holotype PE!, isotypes PE!) (Figs 2 and 3).

**Diagnosis.** *Aster tianmenshanensis* differs from *Aster salwinensis* by its glabrous leaf margins (versus ciliate in *A*. *salwinensis*), unequal disc floret lobes (versus equal), 1-seriate pappus (versus 4-seriate); it differs from *Aster verticillatus* by its unbranched stems (versus branched in *A*. *verticillatus*), larger capitula (15–20 mm in diameter) (versus 4–5 mm), beakless achenes (versus beaked), persistent pappus (versus caducous and often absent).

*Perennial* soboliferous. *Rhizomes* slightly woody, ca. 4–5 mm in diameter. *Stem* solitary, rarely 2 together, unbranched, ca. 10 cm high (including inflorescence), slender, glabrous to shortly pubescent, with many basal rosette leaves and few cauline leaves. *Leaves* of rosette rather thick, sessile, spatulate, 1–5 × 0.5–1 cm, base gradually narrowing, margins slightly revolute, in the distal half of the leaves 1–4-coarsely-serrate, apex acute to slightly obtuse, mucronulate, glabrous or slightly puberulent on both surfaces, with one abaxially prominent main vein and several lateral veins; cauline leaves few, 2–5, present at anthesis, often smaller, the uppermost very small, spatulate to linear, 1–5 × 0.1–1 mm, margins few-serrate to entire. *Capitula* solitary and terminal, 15–20 mm in diameter; involucre campanulate, ca. 6 × 4 mm; phyllaries in 2–3 imbricate series, green, the outer shorter than the inner, lanceolate to elliptic, 3.5–6.0 × 1–1.5 mm, margins more or less scarious, fimbriate-ciliate, abaxially sparsely puberulous, apex acute, receptacle slightly convex. *Ray florets* female, 10–17 with a pubescent tube 2 mm long, ligules white, broadly lanceolate to narrowly elliptic, 8–9 × 2–3 mm, apex slightly retuse with 2 or 3 teeth. *Disc florets* hermaphrodite, many, corolla yellow, 4 mm long, tube 1–1.5 mm, stipitately glandular in the proximal half, 5 lobes triangular, unequal, with one sinus slightly deeper than others, apically slightly glandular. *Achenes* of both floret types identical, oblong, slightly compressed, ca. 1.5 mm long, strigose. *Pappus* 1-seriate, white, barbellate bristles 3–4 mm long, nearly equal to disc corolla at anthesis.

**Phenology.** Flowering from June to July; mature achenes were observed in August and September.

**Distribution, habitat and ecology.** *Aster tianmenshanensis* is known only from Mt. Tianmen, Zhangjiajie City, Hunan Province, China. The climate here belongs to subtropical monsoon, which is cool, foggy and humid (annual rainfall ca.1770 mm) [29]. The new species grows on limestone cliffs at an altitude of ca.1400 m. The cliff tops are covered by mixed evergreen-deciduous forest (Fig 3A) [29]. The only known population is restricted to crevices of the limestone cliffs, where the soil is infertile (Fig 3B) [29]. Another species occupying the same habitat is *Oresitrophe rupifraga* Bunge (Saxifragaceae), which is a newly recorded species in Hunan Province. Recent field collections have revealed other newly recorded species or new species in this area [30–31].

**Systematic position.** The results of our molecular phylogenetic analysis showed that the new species had close relationship with *Aster verticillatus*, *A*. *baccharoides* Steetz and *A*. *turbinatus* S. Moore. All of them were treated as members of *Aster* sect. *Aster* in *Flora of China*. However, our analysis and others [12] revealed that the section was not a monophyletic group. More detailed studies are needed to redefine its circumscription. Here we tentatively placed this new species in *Aster* section *Aster* (Fig 1).

**Conservation status.** *Aster tianmenshanensis* has a very narrow distribution, within that there is only one population with ca. 100 individuals. Despite many surveys of neighboring areas with similar habitats, only the one population has been found. We estimate its distribution area to be less than 10 km^2^. Now a tourist plank walkway passes through its location and its habitat could be easily disturbed or destroyed. This species should be treated as Critically Endangered based on the International Union for Conservation of Nature Red List Categories and Criteria B2a [32].

**Etymology.** The species is named after its type locality, Mt. Tianmen, Hunan Province, China.

**Additional specimens examined (Paratypes).** CHINA. Hunan Province, Zhangjiajie City, Mt. Tianmen, alt. 1400 m, 110° 28´ E, 29° 3´ N, 2 June 2010, *C*. *F*. *Zhang 2720* & *2726* (PE).

#### Key to distinguish *Aster tianmenshanensis* from its most similar species

1. Capitula small, 4–5 mm in diameter; achenes beaked; pappus caducous and often absent..........................................................................................*A*. *verticillatus*
1. Capitula large, more than 10 mm in diameter; achenes beakless; pappus persistent
  2. Stems villous or pilose; leaves villous or pilose, margins ciliate; pappus brownish
    3. Stems ascending, arising laterally from the base of leaf rosettes; stems and leaves eglandular; phyllaries of outer series herbaceous overall..........................................................................................*A*. *salwinensis*
    3. Stems erect, arising centrally from leaf rosettes; stems and leaves glandular; phyllaries of outer series herbaceous above, hardened at base..............................................................................*A*. *fanjingshanicus*
  2. Stems shortly pubescent; leaves glabrous or shortly puberulent, margins not ciliate; pappus white..........................................................................***A*. *tianmenshanensis***

**Nomenclature.** The electronic version of this article in Portable Document Format (PDF) in a work with an ISSN or ISBN will represent a published work according to the International Code of Nomenclature for algae, fungi, and plants, and hence the new names contained in the electronic publication of a PLOS ONE article are effectively published under that Code from the electronic edition alone, so there is no longer any need to provide printed copies. In addition, new names contained in this work have been submitted to IPNI, from where they will be made available to the Global Names Index. The IPNI LSIDs can be resolved and the associated information viewed through any standard web browser by appending the LSID contained in this publication to the prefix http://ipni.org/. The online version of this work is archived and available from the following digital repositories: PubMed Central, LOCKSS.

### Hidden biodiversity on limestone cliffs

*Aster tianmenshanensis* grows in crevices of limestone cliffs (Fig 3A–3C), where there is only a little, infertile soil (Fig 3B) [29]. It is very short and small in habit compared to the other *Aster* species found in southern China. This unique habit may be a consequence of habitat specialization. The special habitat also made it impossible to be encountered until a plank walkway was built across the cliff face for the benefit of tourists. The finding of the new species indicates the lack of our investigation about biodiversity on limestone cliffs. More new species or even new genera probably would be discovered here in the future. Considering the new species’ rarity and special habitat, it’s well worthwhile to explore what mechanisms (e.g. seed and pollen dispersal) allow it to live in such a specialized habitat. Thus, the hidden biodiversity on inaccessible limestone cliffs may provide us with another opportunity to understand the extraordinary life on earth.

## Supporting Information

S1 Table Taxa sampled and their GenBank accession numbers for the ITS, ETS and *trnL-F* sequences used in this study. (DOCX)
