# Supplementary material for: Inaccessible Biodiversity on Limestone Cliffs: Aster tianmenshanensis (Asteraceae), a New Critically Endangered Species from China
Source: PLoS One. 2015 Aug 26;10(8):e0134895. doi: 10.1371/journal.pone.0134895 (PMC4550265; doi:10.1371/journal.pone.0134895)
Supplement: S1 Table — (DOCX) [file pone.0134895.s001.docx]

**S1 Tabel. Taxa sampled and their GenBank accession numbers for the ITS, ETS and *trnL-F* sequences used in this study.**

|  |  | GenBank accessions | | |
| --- | --- | --- | --- | --- |
| Taxon | Voucher or reference | ITS | ETS | *trnL-F* |
| *Arctogeron gramineum* (L.) DC. | Li et al. (2012)  *WPL 0606014* (HNNU) | JN315928 | JN315952 | JN315904 |
| *Aster ageratoides* Turcz. | Li et al. (2012)  *WPL 0112018* (HNNU) | JN543781 | JN543782 | JN543783 |
| *Aster albescens* (DC.) Wall. ex Hand.-Mazz. | Li et al. (2012)  *WPL 0508123* (HNNU) | JN543862 | JN543863 | JN543864 |
| *Aster alpinus* L. | Li et al. (2012)  *WPL 0607020* (HNNU) | JN543817 | JN543818 | JN543819 |
| *Aster altaicus* Willdenow | Li et al. (2012)  *WPL 0506010* (HNNU) | JN543709 | JN543710 | JN543711 |
| *Aster amellus* L. | Li et al. (2012)  *WPL 0408002* (HNNU) | JN543742 | JN543743 | JN543744 |
| *Aster argyropholis* Hand.-Mazz. | Li et al. (2012)  *WPL 0409045* (HNNU) | JN543793 | JN543794 | JN543795 |
| *Aster asteroides* (DC.) Kuntze | Li et al. (2012)  *WPL 0708112* (HNNU) | JN543841 | JN543842 | JN543843 |
| *Aster auriculatus* Franch**.** | Li et al. (2012)  *WPL 0509059* (HNNU) | JN543754 | JN543755 | JN543756 |
| *Aster baccharoides* (Benth.) Steetz | Li et al. (2012)  *WPL 0802001*(HNNU) | JN543805 | JN543806 | JN543807 |
| *Aster batangensis* Bureau & Franch**.** | Li et al. (2012)  *WPL 0606039* (HNNU) | JN543859 | JN543860 | JN543861 |
| *Aster brachytrichus* Franch**.** | Li et al. (2012)  *WPL 0607075* (HNNU) | JN543838 | JN543839 | JN543840 |
| *Aster crenatifolius* Hand.-Mazz. | Li et al. (2012)  *WPL 0409037* (HNNU) | JN543712 | JN543713 | JN543714 |
| *Aster diplostephioides* (DC.) C.B.Clarke | Li et al. (2012)  *WPL 0507020* (HNNU) | JN543847 | JN543848 | JN543849 |
| ***Aster dolichopodus* Y. Ling** | CHINA: Sichuan Province, Lixian county, *Z. X. Fu 70* (PE) | KP313688* | KP313701* | KP313714* |
| *Aster falcifolius* Hand.-Mazz. | Li et al. (2012)  *WPL 0410050* (HNNU) | JN543802 | JN543803 | JN543804 |
| *Aster fanjingshanicus* Y. L. Chen & D. J. Liu | Li et al. (2012)  *WPL 0606082* (HNNU); CHINA: Guizhou Province, Mt. Fanjing, *Fanjingshan Exped. 350* (PE)** | JN543829 | JN543830 | JN543831 |
| *Aster flaccidus* Bunge | Li et al. (2012)  *WPL 0607026*(HNNU) | JN543844 | JN543845 | JN543846 |
| *Aster fuscescens* Bur. & Franch. | Li et al. (2012)  YGS1007021 (HNNU) | JN543751 | JN543752 | JN543753 |
| *Aster handelii* Onno | Li et al. (2012)  *WPL 0708174* (HNNU) | JN543820 | JN543821 | JN543822 |
| *Aster hersileoides* C.K.Schneid. | Li et al. (2012)  *WPL 0807002* (HNNU) | JN543787 | JN543788 | JN543789 |
| *Aster heterolepis* Hand.-Mazz. | Li et al. (2012)  *WPL 0507004* (HNNU) | JN543823 | JN543824 | JN543825 |
| *Aster homochlamydeus* Hand.-Mazz. | Li et al. (2012)  *WPL 0508004* (HNNU) | JN543784 | JN543785 | JN543786 |
| *Aster incisus* Fisch. | Li et al. (2012)  *WPL 0609107* (HNNU) | JN543721 | JN543722 | JN543723 |
| *Aster indicus* L. | Li et al. (2012)  *WPL 0806017* (HNNU) | JN543715 | JN543716 | JN543717 |
| *Aster lavandulifolius* Hand.-Mazz. | Li et al. (2012)  *WPL 0708053* (HNNU) | JN543796 | JN543797 | JN543798 |
| *Aster maackii* Regel | Li et al. (2012)  *WPL 0609043*(HNNU) | JN543745 | JN543746 | JN543747 |
| *Aster nitidus* C. C. Chang | Li et al. (2012)  *WPL 0505007* (HNNU) | JN543790 | JN543791 | JN543792 |
| *Aster oreophilus* Franch. | Li et al. (2012)  *WPL 0509016* (HNNU) | JN543826 | JN543827 | JN543828 |
| *Aster panduratus* Nees ex Walp. | Li et al. (2012)  *WPL 1012067* (HNNU) | JN543757 | JN543758 | JN543759 |
| *Aster pekinensis* (Hance) F. H. Chen | Li et al. (2012)  *WPL 0609077* (HNNU) | JN543718 | JN543719 | JN543720 |
| ***Aster piccolii* Hook.f.** | CHINA: Shanxi Province, Taibai county, *Z. X. Fu 153* (PE) | KP313686* | KP313699* | KP313712* |
| *Aster poliothamnus* Diels | Li et al. (2012)  *WPL 0506001* (HNNU) | JN543763 | JN543764 | JN543765 |
| ***Aster procerus* Hemsl.** | CHINA: Zhejiang Province, Mt. Tianmu, *Z. X. Fu 693* (PE) | KP313683* | KP313696* | KP313709* |
| Aster pycnophyllus Franch. ex Diels | Li et al. (2012)  *WPL 0509091* (HNNU) | JN543799 | JN543800 | JN543801 |
| ***Aster salwinensis* Onno** | CHINA: Xizang Province, Bomi county, *Z. X. Fu 1620* (PE); CHINA: Yunnan Province, Gongshan county, *Z. X. Fu 1699* (PE)** | KP313689* | KP313702* | KP313715* |
| ***Aster savatieri* Makino** | JAPAN: Kumamoto prefecture, *A. Soejima 1* (PE) | KP313687* | KP313700* | KP313713* |
| *Aster scaber* Thunberg | Li et al. (2012)  *WPL 0108025* (HNNU) | JN315934 | JN315958 | JN315910 |
| *Aster setchuenensis* Franch**.** | Li et al. (2012)  *WPL 0508007* (HNNU) | JN543850 | JN543851 | JN543852 |
| *Aster sikuensis* W.W.Sm. & Farrer | Li et al. (2012)  *WPL 0510025* (HNNU) | JN543766 | JN543767 | JN543768 |
| ***Aster sinoangustifolius* Brouillet, Semple & Y. L. Chen** | CHINA: Zhejiang Province, Tiantai County, *Z. X. Fu 189* (PE) | KP313684* | KP313697* | KP313710* |
| ***Aster smithianus* Hand.-Mazz.** | CHINA: Sichuan Province, Maerkang county, *Z. X. Fu 143* (PE) | KP313685* | KP313698* | KP313711* |
| *Aster souliei* Franch**.** | Li et al. (2012)  *WPL 0708084* (HNNU) | JN543835 | JN543836 | JN543837 |
| *Aster taliangshanensis* Y. Ling | Li et al. (2012)  *WPL 0607056* (HNNU) | JN543772 | JN543773 | JN543774 |
| *Aster tataricus* L.f**.** | Li et al. (2012)  *WPL 0108018* (HNNU) | JN543748 | JN543749 | JN543750 |
| ***Aster tianmenshanensis* G. J. Zhang, & T. G. Gao** | CHINA: Hunan Province, Mt. Tianmen, 1, *C. F. Zhang 2720* (PE)**; 2, *C. F. Zhang 2718* (PE); 3, *C. F. Zhang 2726* (PE) | KP313678*; KP313677*; KP313679* | KP313691*; KP313690*; KP313692* | KP313704*; KP313703*; KP313705* |
| *Aster tongolensis* Franch**.** | Li et al. (2012)  *WPL 0708147* (HNNU) | JN543832 | JN543833 | JN543834 |
| *Aster turbinatus* S.Moore | Li et al. (2012)  *WPL 0110029* (HNNU) | JN543814 | JN543815 | JN543816 |
| *Aster verticillatus* (Reinwardt) Brouillet, Semple & Y. L. Chen | Li et al. (2012)  1, CHINA: Yunnan Province, Xichou County, *T. Yang et al. 18266*; *2*, CHINA: Xizang Province, *Tibet Exped. 1022* (PE); 3, *WPL 0607065* (HNNU); CHINA: Chongqing Province, Chengkou County, *T. L. Dai 103080* (PE)** | KP313680*; KP313681*; JN543706 | KP313693*; KP313694*; JN543707 | KP313706*; KP313707*; JN543708 |
| *Aster* *vestitus* Franch**.** | Li et al. (2012)  *WPL 0509023* (HNNU) | JN543769 | JN543770 | JN543771 |
| *Aster* *yunnanensis* Franch. | Li et al. (2012)  *WPL 0508089* (HNNU) | JN543853 | JN543854 | JN543855 |
| *Asterothamnus* *centraliasiaticus* Novopokr**.** | Li et al. (2012)  *WPL 0607045* (HNNU) | JN315930 | JN315954 | JN315906 |
| *Asterothamnus* *fruticosus* (C.Winkl.) Novopokr**.** | Li et al. (2012)  *WPL 0607005* (HNNU) | JN315929 | JN315953 | JN315905 |
| *Bellis* *perennis* L**.** | Li et al. (2012)  *WPL 1003008* (HNNU) | JN315918 | JN315942 | JN315894 |
| *Callistephus* *chinensis* (L.) Nees | Li et al. (2012)  *WPL 0108021* (HNNU) | JN315931 | JN315955 | JN315907 |
| *Chrysanthemum* *indicum* L**.** | Li et al. (2012)  *WPL 1012002* (HNNU) | JN315940 | JN315964 | JN315916 |
| *Conyza* *sumatrensis* (S.F.Blake) Pruski & G.Sancho | Li et al. (2012)  *WPL 1009002* (HNNU) | JN315923 | JN315947 | JN315899 |
| *Crinitina linosyris* (L.) Soják | Li et al. (2012)  *WPL 0408001* (HNNU) | JN315932 | JN315956 | JN315908 |
| *Dichrocephala* *auriculata* (Thunb.) Druce | Li et al. (2012)  *WPL 0708234* (HNNU) | JN315919 | JN315943 | JN315895 |
| *Erigeron* *annuus* (L.) Pers**.** | Li et al. (2012)  *WPL 1010009* (HNNU) | JN315924 | JN315948 | JN315900 |
| *Galatella* *dahurica* DC**.** | Li et al. (2012)  *WPL 0609047* (HNNU) | JN315935 | JN315959 | JN315911 |
| *Grangea* *maderaspatana* (L.) Poir**.** | Li et al. (2012)  *WPL 0802034* (HNNU) | JN315920 | JN315944 | JN315896 |
| *Myriactis* *nepalensis* Less**.** | Li et al. (2012)  *WPL 0509002* (HNNU) | JN315921 | JN315945 | JN315897 |
| ***Nannoglottis yunnanensis* Hand.-Mazz.** | CHINA: Yunnan Province, Yulong county, *T. G. Gao 4807* (PE) | KP313682* | KP313695* | KP313708* |
| *Rhinactinidia eremophila* (Bunge) Novopokr. ex Botsch**.** | Li et al. (2012)  *WPL 0607036* (HNNU) | JN543727 | JN543728 | JN543729 |
| *Rhinactinidia limoniifolia* (Less.) Novopokr. ex Botsch**.** | Li et al. (2012)  *WPL 0607012* (HNNU) | JN543724 | JN543725 | JN543726 |
| *Sheareria* *nana* S.Moore | Li et al. (2012)  *WPL 0701001* (HNNU) | JN543703 | JN543704 | JN543705 |
| *Solidago* *decurrens* Lour**.** | Li et al. (2012)  *WPL 0510116* (HNNU) | JN204176 | JN204177 | JN204178 |
| *Symphyotrichum novi-belgii* (L.) G.L.Nesom | Li et al. (2012)  *WPL 0606002* (HNNU) | JN315926 | JN315950 | JN315902 |
| *Tripolium pannonicum* (Jacq.) Dobrocz**.** | Li et al. (2012)  *WPL 0311001* (HNNU) | JN315937 | JN315961 | JN315913 |
| *Turczaninowia* *fastigiata* (Fisch.) DC**.** | Li et al. (2012)  *WPL 0609030* (HNNU) | JN543739 | JN543740 | JN543741 |

* Sequences generated in this study (taxon names in bold).

** Accessions for micromorphological observations.
